# Supplementary material for: Design, development, and characterization of amorphous rosuvastatin calcium tablets
Source: PLoS One. 2022 Mar 21;17(3):e0265263. doi: 10.1371/journal.pone.0265263 (PMC8936501; doi:10.1371/journal.pone.0265263)
Supplement: Attachment — Submitted filename: Response to reviewers.docx [file pone.0265263.s001.docx]

Thank you very much for your comments and suggestions concerning the manuscript PONE-D-21-36028 entitled **“Design, development, and characterization of amorphous rosuvastatin calcium tablets”.** These comments are very valuable and play a key role in revising and improving the manuscript. We have carefully studied the comments and made corrections.

We sincerely hope to get your approval. The revised part was marked in yellow on the manuscript. The main corrections in this article and the response to the reviewer’s comments are as follows:

**Reviewer #1:**

1. Regarding the r value, how it was calculated. An explanation on this issue has been added on page 6.
2. Also, regarding the r value presented in table 2, I think that fig. 11 represent these values and not figure 3, please check. It is correct.
3. According, to USP, it is recommended to make a content uniformity for tablets containing less than 25 mg of active ingredients and calculates the acceptance value. Did the author follow this requirement, and if so, it is better to add the AV beside the weight variation data. Content uniformity studies have been added. new parts have been added to discuss the results obtained regarding the content uniformity study.

**Reviewer #2:**

1. The manuscript is overall written in an easy-to-read way, and it is highly appreciated. However, the introduction section is suggested to be slightly reduced for the sake of conciseness. This section has been slightly shortened.
2. Apart from that, the results section should preferably be renamed as results and discussion (as results are partly discussed already there) so that current discussion can be renamed as conclusions. The results section has been renamed to results and discussion.
3. Lastly, Pharmacopeia methods should rather be specified in the main body of the text than be referenced at the end of the article as any other citation, as in this view it is much clear which experiments have been conducted following Pharmacopeia's procedures. In relation to the methods of the Pharmacopoeia, they have been specified in the main body of the text, as you well suggest.
